# Inverse Shifting-PCR Modified by Capillary Electrophoresis for Detecting F8 *int22h* and *int1h* Inversions in Severe Hemophilia A Patients and Probable Carriers

**DOI:** 10.3390/life14101332

**Published:** 2024-10-18

**Authors:** Rosa Michel Martínez-Contreras, Silvia Sofía García-López, Hilda Luna-Záizar, Ana Rebeca Jaloma-Cruz

**Affiliations:** 1 Doctorado en Genética Humana, Centro Universitario de Ciencias de la Salud, Universidad de Guadalajara, Guadalajara 44340, Jalisco, Mexico; michelmartinezc@gmail.com; 2 Facultad de Biología, Universidad Autónoma de Sinaloa, Culiacán 80010, Sinaloa, Mexico; sojy.9@hotmail.com; 3 Departamento de Química, Centro Universitario de Ciencias Exactas e Ingenierías, Universidad de Guadalajara, Guadalajara 44430, Jalisco, Mexico; hiluna90@yahoo.com.mx; 4 División de Genética, Centro de Investigación Biomédica de Occidente, Instituto Mexicano del Seguro Social, Guadalajara 44340, Jalisco, Mexico

**Keywords:** factor VIII, severe hemophilia A, intron 1 and 22 inversions, fluorescent capillary electrophoresis, inverse shifting-polymerase chain reaction

## Abstract

Globally, intron 22 inversions (Inv22s) of the factor VIII gene (F8) are the most frequent pathogenic variants and account for 45–50% of severe hemophilia A (SHA) cases, while intron 1 inversion (Inv1) explains 1–5% of SHA cases. The detection of both inversions by an inverse shifting-polymerase chain reaction (IS-PCR) is the first choice worldwide for the diagnosis of patients and carriers of SHA. To improve its sensitivity and reproducibility in the visualization of PCR products, we approached the IS-PCR with fluorescent capillary electrophoresis instead of agarose electrophoresis. Based on the original protocol, we modified two primers by 5’-end labeling with FAM™ fluorescent dye for the detection of the PCR products by capillary electrophoresis. Additionally, the “fast enzymes” *Bcl*I and T4-Ligase were incorporated for work saving in the genomic digestion and ligation reactions, respectively. Once we accomplished the standardization and verified the reproducibility of the modified IS-PCR method, we applied it for the diagnosis of a cohort of SHA patients and carriers. The modified IS-PCR by fluorescent capillary electrophoresis for PCR product detection is more sensitive than agarose electrophoresis. The method was also improved by using the new rapid enzymes to save time in the whole process.

## 1. Introduction

Hemophilia A (HA) is an X-linked disorder characterized by the functional deficiency of coagulation factor VIII (FVIII) resulting from pathogenic variants in the FVIII gene (F8); it has a worldwide incidence of 1/5000 male births [1].

The most frequent causes of severe hemophilia A (SHA) are large intron 22 inversions (40–50%) [2] and intron 1 inversions (1–5%) in the F8 gene [3]. These complex rearrangements occur during male meiosis via a homologous intrachromosomal recombination between repeats within the F8 gene and their telomeric homologues [4].

Intron 22 inversions (Inv22s) are generated by the recombination between the intron 22 homologous region 1 (*int22h-1*), located in the F8 *locus*, and either of its homologous extragenic copies, *int22h-2* (Inv22 type 2) or *int22h-3* (Inv22 type 1), located at 400 kb and 500 kb toward the telomere, respectively [5]. These rearrangements cause the inversion and recombination of exons 1 to 22 at the telomeric region, breaking up from exons 23 to 26 in the F8 *locus*, and ultimately impair the production of the FVIII protein whose deficiency causes the manifestation of SHA [5].

Analogously, the intron 1 breaking inversion arises from a recombination between a repeated region of 1041 kb in *int1h-1* and the highly homologous extragenic region *int1h-2* located at 140 kb from the F8 gene [3].

The detection of these complex rearrangements has been a technical challenge since their identification nine years after the full sequencing of the F8 gene in 1984 [2]. Thus, the main methods used so far are Southern blot, long-distance PCR (LD-PCR), and inverse shifting-PCR (IS-PCR). Their development and technical details have been described in a comprehensive analysis that highlights the challenges and international collaboration involved [6].

Currently, IS-PCR is the most widely used method for inversion detection worldwide, even though the visualization of amplified fragments on an agarose gel is occasionally difficult. We have used this robust method in a large cohort of patients and families with SHA [7] and experienced some problems in detecting PCR products on agarose gels. Therefore, we took advantage of the improved sensitivity that capillary electrophoresis could offer [8] and tested fluorescent labeling on common primers for the detection of Inv22 and Inv1, as reported by Rossetti et al. [9]. This approach significantly enhanced the reproducibility of results, and the use of rapid enzymes reduced the time needed for the procedure.

## 2. Materials and Methods

We conducted a cohort study of Mexican SHA patients and their families from 11 states of the country who gave their consent to participate from 2021 to date.

### 2.1. Characteristics of Participants and Description of Materials

The SHA patients were diagnosed by FVIII level assessed by a one-stage clotting assay according to the suppliers in the respective clinical laboratory of each patient’s hospital.

The patients were referred by their hematologists to our laboratory for genetic diagnosis. The adult patients, or the mentors in the case of pediatric individuals, gave their consent to participate in the study. We collected their blood samples by venopunction to obtain genomic DNA from peripheral blood leucocytes by salting out and digestion with Proteinase K followed by precipitation with NaCl 6M, ethanol washes, and resuspension in Tris-EDTA buffer according to the original protocol [10]. The DNA was quantified in NanoDrop™ 2000/2000c-(Thermo Fisher Scientific™, Waltham, MA, USA) describe, and stored at −20 °C until its use.

### 2.2. Processes, Interventions, and Comparisons

Inv22s and Inv1 were detected by IS-PCR [9], following the processing procedure from a former publication [11] and complemented with long-capillary electrophoresis, based on the method reported by Pan et al. [7] except for minimal modifications in the reactants’ brands, reaction volumes, or types of purification methods for the digestion and ligation products (Table 1). We used the set of primers of the original IS-PCR [9] but labeled two primers at the 5′-end with the fluorescent dye FAM™ to detect the PCR products by capillary electrophoresis in the ABI-Prism ^®^ 310 sequencer (Applied Biosystems©, Foster City, CA, USA), and analyzed them using the Peak-Scanner ^®^ v1.0 free access software. The programed conditions of the capillary electrophoresis were a temperature of 60 °C, a sample injection at 15 kV for three seconds, and 28 min of running; 500 LIZ™ was used as a molecular marker.

### 2.3. Statistical Analysis

We used the statistical software SPSS v.21 (IBM Corp, Armonk, NY, USA) for descriptive statistics.

Comparative flowcharts are presented to identify the components that we considered from each author, and the novel elements that we added are in bold.

## 3. Results

We accomplished the standardization and verified the reproducibility of the modified IS-PCR method coupled with fluorescent capillary electrophoresis by testing inversion-negative and -positive patients and carriers who were previously validated by agarose electrophoresis and are usually used as control samples for all assays in our laboratory.

**Figure 1:**
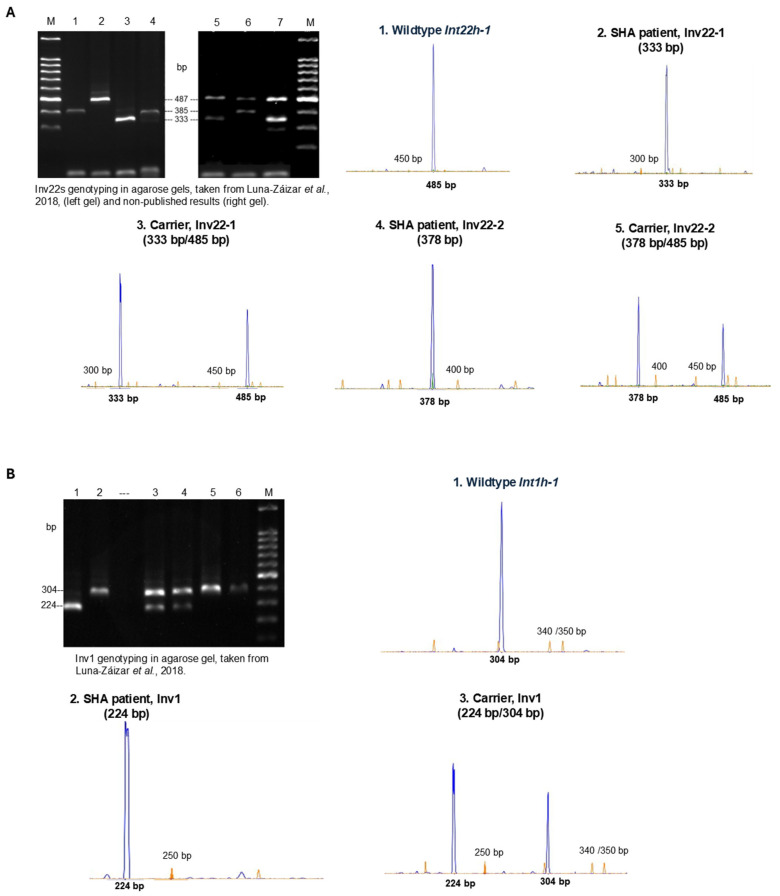
Genotyping of Inv22 (**A**) and Inv1 (**B**) by IS-PCR contrasting the results of agarose gels and fluorescent capillary electrophoresis. Gel agarose pictures were taken from a previous study by our group, Luna-Záizar et al., 2018 [7] and from non-published results, based on the protocol of Rossetti et al., 2008 [9]. The panel numbers of the PCR products observed as fluorescent peaks represent similar genotyping results to the corresponding diagnosed cases in the agarose gel. The PCR products are observed as peaks 5′-end-labeled in blue by 56-FAM™ and the molecular ladder (minor peaks) is labeled in orange by 500 LIZ™; fragment sizes are indicated in base pairs (bp).

Figure 1 shows the genotypes of the normal repeats *int22-h1*, *int1-h1*, and related inversions in SHA patients and carriers as revealed by IS-PCR and fluorescent capillary electrophoresis. These data are compared with the previous genotyping in agarose gel we obtained [7] using the original IS-PCR method of Rossetti et al. [9].

**A. Genotyping of Inv22-1 and Inv22-2 in agarose gel.** Molecular marker (M), 100 bp DNA ladder; left gel: lanes (1,4) Inv22-2 SHA patient; (2) non-Inv22, wildtype (WT); (3) Inv22-1 SHA patient; right gel: (5,7) Inv22-1 carrier; (6) Inv22-2 carrier. The size of the PCR products is indicated in bp.

**Inv22 diagnostic test modified by fluorescent capillary electrophoresis.**

1. *Int22-h1*, WT_FAM 485 bp; LIZ 450 bp.
2. Inv22-1 SHA patient_FAM 333 bp; LIZ 300 bp.
3. Inv22-1 carrier_FAM 333 bp; LIZ 300 bp/FAM 485 bp; LIZ 450 bp.
4. Inv22-2 SHA patient_FAM 378 bp; LIZ 400 bp.
5. Inv22-2 carrier_FAM 378 bp; LIZ 400 bp/FAM 485 bp; LIZ 500 bp.

**B. Genotyping of Inv1 in agarose gel.** Lane (1) Inv1 SHA patient; (2,5,6) non-Inv1, wildtype (WT); (3,4) Inv1 carrier; molecular marker (M), 100 bp DNA ladder. The size of the PCR products is indicated in bp.

**Inv1 diagnostic test modified by fluorescent capillary electrophoresis.**

1. *Int1-h1*, WT_FAM 304 bp; LIZ 340/350 bp.
2. Inv1 SHA patient, FAM 224 bp; LIZ 250 bp.
3. Inv1 carrier, FAM 224 bp; LIZ 250 bp/FAM 304 bp; LIZ 340/350 bp.

We studied 36 unrelated Mexican SHA patients and 42 of their female relatives. Among the former, 16 had a family history of hemophilia and 20 were sporadic cases, while 24 were children or teenagers (2–17 years old) and 12 were adults (18–45 years old). Regarding geographical origin, 15 patients (42%) were from Jalisco while the remaining 21 (58%) came from 10 other states of the country.

Twenty-two patients (61.1%) were positive for Inv22s, eighteen carried Inv22-1 and four carried Inv22-2; among the fourteen cases negative for Inv22s, two patients were positive for Inv1 (2/36, 5.6%) and twelve SHA patients were negative for both inversions (33.3%).

For the carrier diagnosis, we first searched for Inv22s in probands and their mothers, either obligate carriers in familial cases or mothers of a sporadic affected son. In those mothers who tested negative, we searched for Inv1. Based on these results, we expanded our search to other probable carriers. Altogether, 33 cases were diagnosed and only two families negative for both inversions were found. We confirmed that most mothers of sporadic patients had at least one of the inversions and were therefore diagnosed as carriers (14 of 15 mothers). For the single family where the mother did not have the Inv22-2 carried by her son, we assumed a de novo pathogenic variant in the patient or germinal mosaicism in the mother.

## 4. Discussion

The search for pathogenic inversions in SHA patients as the first diagnostic option is a very valuable resource to provide medical care to SHA patients and genetic diagnoses to their families, mainly in developing countries like Mexico where sequencing tools are not available in public health institutions. Our cohort showed a frequency of inversions of 61%, which is higher than the 45–50% ratio observed in different populations of SHA patients [2], including a previously studied cohort of Mexican patients (47.2%) [12]. However, our sample is biased because of the predominance of pediatric patients (66%) and sporadic cases (55%), i.e., it does not represent the general population of SHA patients.

Regarding SHA patients negative for both inversions, the genetic diagnosis requires alternative approaches like MLPA and Sanger or exome sequencing [13], which are necessary to fully assess the spectrum of pathogenic variants and the genotype–phenotype correlation [13]. This is especially relevant in the detection of liveborn carriers and in prenatal or preimplantation diagnoses [14].

The results here presented, fully agree with those obtained via the original IS-PCR protocol and the visualization of products in agarose gels dyed with SybrGreen^®^ [7]. The results reported by Pan et al. [7], with the same modified IS-PCR that shows higher sensitivity and resolution than agarose gel electrophoresis for Inv22s genotyping, encouraged us to test such an approach under our conditions, employing the GeneScan™ application of the ABI PRISM^®^ 310 Genetic Analyzer.

Our genotyping results were clearer and with specific peaks (instead of fragments) of the expected sizes for the corresponding PCR products. The occasional discordance of 2–5 bp from the product size determined by the original protocol [9] is unsurprising because the known effect of fluorescent dyes on the electrophoretic mobility of PCR products [15].

We noted that the PCR products corresponding to Inv22-2 of 385 bp, and wildtype *Int22h-1* of 487 bp, according to the original PCR protocol [9], were observed as peaks of 378 bp and 485 bp, respectively (Figure 1).

In addition to being highly reproducible, our genotyping results showed the sizes expected for wildtype and inversions with minimal variability (±1–2 bp) among different assays. These trivial discrepancies can be ascribed to differences in the run temperature, gel polymer, or running buffers. Noticeably, our results were consistent and reproducible with different amounts of ligation product in the PCR reaction and in different experiments with the same sample.

As for cost effectiveness, we estimate a 3-fold higher cost for the IS-PCR method that uses capillary electrophoresis instead of agarose gels. However, the detection of inversions by the latter is sometimes difficult because of “smiling” and other artifacts in the running gel or faint signals of the expected bands related to different yields of the PCR ligation products. This has been especially critical for Inv22-2 due to lower PCR yields that may even require a repeat of the PCR or the whole process, which takes 3–4 days to be completed.

The original IS-PCR protocol of Rossetti et al., 2008 [9], designed with basic methods of molecular biology, can be used worldwide as a screening program to detect the most common pathogenic variants in the hemophilia population.

Our modification with capillary electrophoresis requires specialized infrastructure, which is generally available in research centers or universities, but is mostly unavailable in the clinical facilities of public health institutions.

Considering the advantages of sensitivity and reliability, our improved method may be implemented in regional centers of reference to diagnose hemophilia and other rare diseases, especially in Mexico and Latin America.

Although we report a minor modification of the original IS-PCR method, our experience might be translated to other laboratories with an availability of new generation capillary sequencers, allowing for further modifications and technical details that could enhance the robustness of the method.

## 5. Conclusions

The IS-PCR modified by fluorescence capillary electrophoresis for the detection of products is more sensitive and reproducible than agarose electrophoresis under our laboratory conditions. The full method also improved the time required by using new rapid enzymes in the genomic digestion and ligation reaction.

## Figures and Tables

**Table 1:** Comparative chart of the reference protocols of IS-PCR considered for the proposed modification with fluorescent capillary electrophoresis. “*” refers to modified primers.

Protocol Step	Rossetti et al, 2005 [11]; 2008 [9]	Pan et al, 2014 [8]	This Study
***Bcl*** **I Digestion**	Genomic DNA, 2 µg, is digested with 20 U *of Bcl*l enzyme (Promega, Madison, WI, USA). Incubation at 50 °C for 4 h, volume reaction of 50 µL	Genomic DNA, 5 µg, is digested with 20 U of *Bcl*І enzyme (New England Biolabs, Beverly, MA, USA), incubation at 50 °C for 2 h, volume reaction of 50 µL	Genomic DNA, 2 µg, is digested with 20 U of ***Bcl*І enzyme (Jena Bioscience, Jena, Germany), incubation at 50 °C for 10 min**, volume reaction of 50 µL
**1st. Purification**	Phenol–chloroform Ethanol precipitation: NaCl 3M, 0.11 volumes, final concentration of 0.3 mol/L with 2 volumes of ethanol. Digested DNA is resuspended in 50 µL of distilled water	Ethanol precipitation. Two washes with ethanol 75% Digested DNA is resuspended in 10 µL of distilled water	Ethanol precipitation **One wash with ethanol 70%** Digested DNA is resuspended in 10 µL of HPLC water
**Ligation**	DNA fragments are self-circularized with 3 U of T4 DNA Ligase (Invitrogen, Buenos Aires, Argentina) in 400 µL of volume reaction at 15 °C, overnight	DNA fragments are self-circularized with 3U of T4 DNA ligase (Takara Biotechnology, Japan) in 100 µL of volume reaction at 15 °C for 12 h	DNA fragments are self-circularized with 3U of **T4 DNA ligase, (Jena Bioscience, Jena, Germany) in 200 µL of volume reaction at 16 °C for 30 min**
**2nd. Purification**	Phenol–chloroform Ethanol precipitation or alternatively: GFX^TM^ Spin chromatography columns (Amersham, Buenos Aires, Argentina). Ligated DNA is recovered in 30 µL of distilled water	Ethanol precipitation Two washes with ethanol 75% Ligated DNA is recovered in 10 μL of sterile water	Ethanol precipitation **One wash with ethanol 70%** Ligated DNA is recovered in 20 μL of HPLC water
**Multiplex PCR**	PCR is performed with 3 µL and 6 µL of circularized DNA for the analysis of Inv1 and Inv22, respectively, in the presence of 0.6 µM of each primer, 0.5 U of Taq DNA Polymerase (Promega^TM^, Buenos Aires, Argentina), and additional standard PCR reagents in a total volume of 25μL. (1) Inv22-diagnostic for a pattern-sensitive detection of deleterious mutations (Inv22 and Del22) from non-deleterious variants (Dup22 and normal); (2) Inv1-diagnostic; and (3) Inv22-complementary for discrimination between Inv22 and Del22, and between Dup22 and normal. Thermocycling: 94 °C for 2 min *Denaturation at 94 °C, 30 s* *Primer annealing at 56 °C, 1 min* *Extension at 72 °C for 90 s, 30 cycles* 5 min at 72 °C	PCR is performed with 200 ng of circularized DNA, 1U of Takara rTaq, and additional standard PCR reagent in a total volume of 25 µL. Five primers were used at 0.6 µM of each primer to perform the diagnostic and complementary test for genotyping all the possible rearrangements of Inv22, including two modified primers* from Rossetti et al., 2008 The final reaction volume was 25µL containing 2. DMD amplification as internal standard (IS). Thermocycling: 95 °C for 10 min *Denaturation at 95 °C, 30 s* *Primer annealing at 57 °C, 1 min* *Extension at 72 °C, for 90 s, 30 cycles* 5 min at 72 °C	PCR is performed with 3 µL and 6 µL of circularized DNA for the diagnostic test for Inv1 and Inv22, respectively. We used the primers described by Rossetti et al. [9] but modified with 5’-end label of fluorescent dyes. **Maxima Hot-Start Polymerase (Thermo Fisher Scientific Inc, Waltham, MA, USA), was used to enhance the specifity, sensitivity and yield of the PCR products.** The PCR program was modified to activate the enzyme according to the manufacturer’ recommendations: Thermocycling: 95 °C for 4 min *Denaturation at **95 °C**, 30 s* *Primer annealing at 56 °C, 1 min* *Extension at 72 °C for 90 s, 30 cycles* 5 min at 72 °C
**Primers**	**Inv22**		
	22-IU CCTTTCAACTCCATCTCCAT	22h-1U AACTCCCTTCCTTGTCAGCA	22-IU CCTTTCAACTCCATCTCCAT
	22-2U ACGTGTCTTTTGGAGAAGTC	22h-2U ACGTGTCTTTTGGAGAAGTC	22-2U ACGTGTCTTTTGGAGAAGTC
	22-3U CTCACATTGTGTTCTTGTAGTC	22h-3U CTCACATTGTGTTCTTGTAGTC	22-3U CTCACATTGTGTTCTTGTAGTC
	22-ID ACATACGGTTTAGTCACAAGT	IPCR-ID ACATACGGTTTAGTCACAAGT	22-ID **FAM** ACATACGGTTTAGTCACAAGT *
	22-ED TCCAGTCACTTAGGCTCAG	IPCR-ED TCCAGTCACTTAGGCTCAG	
	**Inv1**		
	1-IU GCCGATTGCTTATTTATATC	--	1-IU **FAM** GCCGATTGCTTATTTATATC*
	1-ID TCTGCAACTGGTACTCATC	--	1-ID TCTGCAACTGGTACTCATC
	1-ED GCCTTTACAATCCAACACT	--	1-ED GCCTTTACAATCCAACACT
**Amplified products**	Inv22/Diagnostic test	Inv22/Diagnostic and complementary tests	Inv22/Diagnostic test
	487 bp, *Int22-h1* WT 333 bp, Inv22-1 385 pb, Inv22-2	Wildtype alleles: 512 bp, *Int22-h1;* 457 bp, *Int22-h2*; 405 bp, *Int22-h3* Inv22-1 related alleles: 333 bp, Inv22-1; 457 bp, *Int22-h2* WT; 584 bp, Dup22-1 *Inv22-2* related alleles: 385 bp, Inv22-2; 405 pb, *Int22-h3* WT; 584 bp, Dup22-2	FAM 485 bp, *Int22-h1*, WT FAM 333 bp, Inv22-1 FAM 378 bp, Inv22-2
	Inv22/Complementary test		Inv22/Complementary test
	Wildtype alleles and duplications 559 bp, Benign Dup22-1 or Dup22-2 457 bp, *Int22-h2* WT, related to Inv22-1, Del22-1 405 bp, *Int22-h3* WT, related to Inv22-2, Del22-2		Not done
	Inv1/Diagnostic test		Inv1/Diagnostic test
	304 bp, *Int1-h1* WT 224 bp, Inv1		FAM 304 bp, *Int1-h1* WT FAM 224 bp, Inv1
**Visualization of amplified products**	Electrophoresis on 1.5–2% agarose gels stained by ethidium bromide	PCR products and IS are 1/5 and 1/10 diluted, respectively. Short-end capillary gel electrophoresis, Polymer prepared with intercalating dye, YO-PROs-1 Iodide, Molecular Probes (Invitrogen^TM^, Eugene, OR, USA)	**Fluorescent capillary electrophoresis performing the GeneScan™** application with the ABI PRISM® 310 Genetic Analyzer, and 500 LIZ™ was used as molecular marker

## Data Availability

All data generated or analyzed during this study are included in this article. The data that support the findings of this study are available on request from the corresponding author. The data are not publicly available due to privacy or ethical restrictions.
